# External Resistances Applied to MFC Affect Core Microbiome and Swine Manure Treatment Efficiencies

**DOI:** 10.1371/journal.pone.0164044

**Published:** 2016-10-04

**Authors:** Anna Vilajeliu-Pons, Lluis Bañeras, Sebastià Puig, Daniele Molognoni, Albert Vilà-Rovira, Elena Hernández-del Amo, Maria D. Balaguer, Jesús Colprim

**Affiliations:** 1 LEQUiA, Institute of the Environment, University of Girona, Girona, Spain; 2 Molecular Microbial Ecology Group, Institute of Aquatic Ecology, University of Girona, Girona, Spain; 3 Department of Civil Engineering and Architecture (D.I.C.Ar.), University of Pavia, Pavia, Italy; University of Illinois at Chicago, UNITED STATES

## Abstract

Microbial fuel cells (MFCs) can be designed to combine water treatment with concomitant electricity production. Animal manure treatment has been poorly explored using MFCs, and its implementation at full-scale primarily relies on the bacterial distribution and activity within the treatment cell. This study reports the bacterial community changes at four positions within the anode of two almost identically operated MFCs fed swine manure. Changes in the microbiome structure are described according to the MFC fluid dynamics and the application of a maximum power point tracking system (MPPT) compared to a fixed resistance system (Ref-MFC). Both external resistance and cell hydrodynamics are thought to heavily influence MFC performance. The microbiome was characterised both quantitatively (qPCR) and qualitatively (454-pyrosequencing) by targeting bacterial 16S rRNA genes. The diversity of the microbial community in the MFC biofilm was reduced and differed from the influent swine manure. The adopted electric condition (MPPT vs fixed resistance) was more relevant than the fluid dynamics in shaping the MFC microbiome. MPPT control positively affected bacterial abundance and promoted the selection of putatively exoelectrogenic bacteria in the MFC core microbiome (*Sedimentibacter* sp. and gammaproteobacteria). These differences in the microbiome may be responsible for the two-fold increase in power production achieved by the MPPT-MFC compared to the Ref-MFC.

## Introduction

Continuous and unsustainable animal production causes an accumulation of undesirable products such as swine manure, which has a complex organic matter and nitrogen (primarily ammonium) content that contributes to environmental pollution. Annually, 1.4 billion tonnes of swine manure are generated in the European Union, where the main contributor country is France [1]. Many technologies have been proposed to treat these pollutants, of which the most commonly employed is anaerobic digestion. However, these processes entail high operation costs, large surface availability and low treatment efficiencies [2,3]. Microbial fuel cells (MFCs) are a newer biological technology for animal wastewater treatment with concomitant electricity production [4,5], and may be able to address limitations of anaerobic digestion.

Interest in MFCs has increased during the last decade, especially regarding their use in scaled-up applications [6–8] for *in situ* swine manure treatment. MFCs will become a recognisable alternative technology for green energy recovery from wastewater treatment in the near future. Scientific reports on MFCs include the evaluation of their nutrient removal capacities [9], power production [10], or microbial community characterization [11], which generally are analysed separately. A simultaneous, multi-disciplinary approach has rarely been performed, although this type of approach may be required to obtain an in-depth understanding and optimize the technology for use.

Microbial communities are essential for the bioelectrochemical processes in MFCs. In the anode chamber, exoelectrogenic microorganisms oxidise organic matter to release electrons to the anode electrode [12]. The exoelectrogenic respiration capacity has been thoroughly studied using model organisms growing on acetate, including *Geobacter sulfurreducens* PCA [13] and *Shewanella oneidensis* MR-1 [14]. The structural and biochemical properties of the different extracellular electron transfer (EET) mechanisms performed by these two strains have been studied [15,16]. However, MFC application to wastewater treatment increases bacterial community complexity, leading to interconnected relationships among the cells that make it difficult to study the exoelectrogenic capacities of each identified strain. In a few studies, identified microorganisms were specifically related to substrate degradation and electricity production in MFCs treating wastewater [17,18]. For example, Velvizhi and Mohan (2015) focused on the identification of EET sites and processes that were linked to the degradation of pharmaceutical wastewater [19].

Additional syntrophic relationships occur in biofilms and may be essential for the degradation of complex organic matrices [20], especially when combined with interspecies electron transfer events [21,22]. The development of structured microbial communities within MFC anodes showed significant advantages compared to pure exoelectrogenic communities in the treatment of complex organic matter matrices, such as urban wastewater [23], feedstock wastewater [24], landfill leachate [25] and more recently swine manure [26]. The reasons for the enhanced performance of complex-structured biofilms relied on the increased resilience of the cells, protection against toxic substances, and closer contact between cells, which might facilitate communication through biochemical signals and assist with nutrient distribution.

New methodologies, such as high-troughput amplicon sequencing [27], PhyloChip analysis [28], flow cytometry [29], and stable isotope probing (SIP) [30], have been applied to identify microorganisms performing specific metabolic processes within complex matrices. In this sense, cultivation independent methods have significantly added to the analysis of relevant taxa that have escaped cultivation so far. However, despite these technical advances, species identification and the determination of their active roles in the biofilm remain difficult. In most cases, indirect comparative methods must be used. Biofilms able to treat complex substrates are usually characterised in terms of the community (microbiome) instead of the individual species. The identification of microbial groups potentially responsible for electricity production in MFCs (i.e., exoelectrogenic groups) can be accomplished by comparing the microbial communities that develop under well-differentiated conditions.

Different control strategies can be applied to improve MFC functionality and enhance the activity of the microorganisms associated with the exoelectrogenic process. Strategies aimed at reaching and maintaining the maximum power point (MPP) of the MFC were developed. The MPP is reached when the MFC internal and external resistances coincide [31]. Its continuous tracking (called MPPT) has achieved several advantages as follows: enhanced exoelectrogenic activity of microorganisms [32], higher electricity production and coulombic efficiency (CE) [33] and reduced side effects, such as methane production [34]. Nevertheless, the relationship between the structure of the microbial community and electricity production in MPPT-controlled MFCs remains unclear.

In addition to MPPT control, the reactor design is a determinant for fluid dynamics and therefore homogeneity and mass transfer kinetics within the cell [35][36]. Computational fluid dynamics methods can be used to study the fluid distribution but rarely have been applied to MFC research. Few studies are available to assist in reactor design and optimization. Kim et al. (2012) studied the effects of the influent flow rate and substrate concentration on MFC performance and concluded that high flow rates (7.5 mL min^-1^) correlated with maximum power production (2.7 mW) [37]. Moreover, a recent study by Michie et al. (2014) showed a 40% increase in bacterial abundance when adopting a turbulent flow regime (shear rate of 237 s^-1^). The microorganism distribution inside the MFCs was not investigated in any of these studies [38].

In this study, different external resistance control strategies were applied to two replicate MFCs fed swine manure. The effect on the development of the exoelectrogenic bacterial community was evaluated to optimise the internal MFC bioprocesses. A MPPT control strategy (based on dynamic resistance) was applied to one of the MFCs and compared with a Ref-MFC operated at fixed resistance. Microbial groups developing in the anode chamber were identified. The comparative analysis of the MFC core microbiomes allowed the identification of bacteria that were potentially responsible for the power generation. Moreover, the internal distribution of the bacterial abundance was analysed and related to the reactor fluid dynamics.

## Materials and Methods

### Experimental set-up and inoculation

Two replicate, dual-chamber MFCs were constructed and operated to remove organic matter from swine manure with bioelectricity production. Details of the MFC design and operation are described in the study of Molognoni *et al*. (2014). Briefly, each MFC consisted of a methacrylate, rectangular reactor with an anode and cathode placed on opposite sides of an Anionic Exchange Membrane (AMI-7001, International Membranes Inc., USA). Each anode and cathode chamber contained approximately 400 mL of liquid volume (S1 File) [39]. Swine manure collected at an experimental station of the Food and Agricultural Research Institute (IRTA, Girona, Spain) was continuously fed to the anode at a flow rate of 1.5 L d^-1^. The continuous replacement of fresh swine manure (main characteristics in S1 Table) maintained the organic loading rate (OLR) at 10.5 ± 0.7 kg COD m^-3^ d^-1^ for the entire experimental period (43 days). The cathode was fed an oxygen-saturated inorganic solution (see Supplementary methods). The temperature was kept constant at 21 ± 1°C.

**Fig 1:**
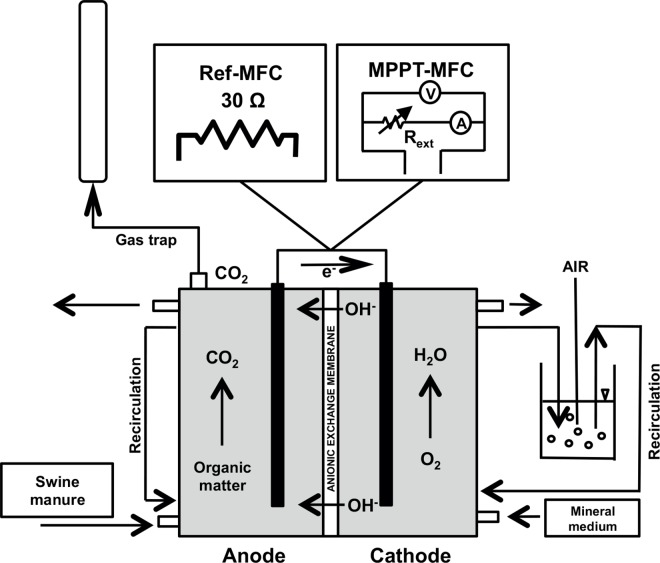
Schematic representation of replicate MFC configurations with the two evaluated electrical load conditions (Ref-MFC and MPPT-MFC).

The two MFCs operated under the same hydraulic conditions and differed only in the electrical load application (Fig 1). The MPPT-MFC operated with an automatically controlled resistance, whereas the Ref-MFC operated at a fixed resistance of 30 Ω. The fixed resistance value was chosen to approximate the MFC internal resistance based on previous experience [26].

The anode and cathode chambers of both MFCs were inoculated as described by Molognoni *et al*. [39]. 2-Bromoethanesulfonate (BES) was added to prevent methanogen growth only during start-up. Four days after inoculation, the MFCs were continuously fed swine manure and the electric control was switched on for the MPPT-MFC.

### Maximum power point tracking control

The MPPT control consisted of an array of parallel-connected potentiometers imposing resistance on the MFC, an amperometer and a voltmeter (Model 2000 6-1/2 Digit Multimeter, Keithley Instruments, USA) for current and voltage measurements [39]. The applied external resistance could vary between 6 and 200 Ω via 2 Ω steps (ΔR). The implemented MPPT algorithm is classified as a perturbation-observation method. Basically, it was composed of a loop that periodically measured the MFC output power (P). The power value measured at one iteration (step i) was compared with the value measured in the previous iteration (step i-1); the resistance R varied according to Eq 1.

$$R_{i+1}=R_{i}+\Delta R\;sign\;\left(\frac{P_{i}\;\text{-}\;P_{i\text{-}1}}{R_{i}\;\text{-}\;R_{i\text{-}1}}\right)$$ (1)

### Computational fluid dynamics

**Fig 2:**
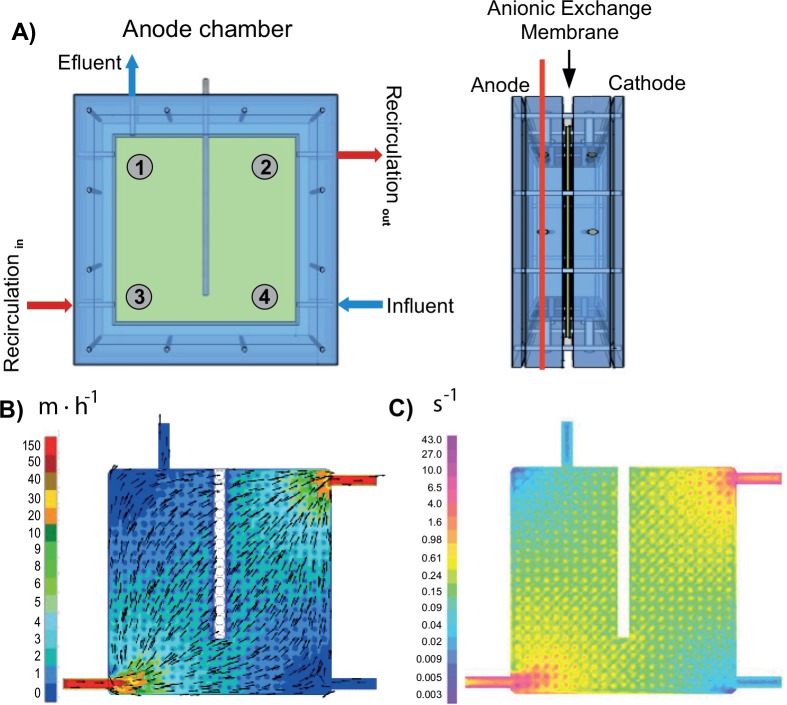
A) Schematic figure of the anode compartment where the 4 samples (1, 2, 3 and 4) for microbial analyses are represented. Hydrodynamic model results in terms of (B) fluid velocity and (C) shear rate profile distribution.

A fluid dynamic model of the MFC anode chamber was developed using the Ansys Fluent software (ANSYS® Academic Research, Release 12.1). The fluid dynamic equations and shear rate calculation were solved in Vilà-Rovira *et al*. [40]. Fig 2 shows the anode chamber configuration scheme together with the fluid velocity and shear rate internal distributions under the MFCs’ operational conditions (influent flow rates of 1.5 L·d^-1^ and recirculation flow rates of 170 L·d^-1^).

### Chemical and electrochemical analyses and calculations

Liquid phase standard wastewater measurements for organic matter, nitrogen and solid content were performed at regular intervals according to the American Public Health Association guidelines [41]. Samples were obtained from the (anode) influent and effluent sections of the MFCs. Detailed calculations of the chemical analysis performed are provided in the supplementary methods (S1 File). Gas samples were analysed to detect the presence of carbon dioxide and methane (CO_2_ and CH_4_) with an Agilent 7820A GC System equipped with the Washed Molecular Sieve 5A and Porapak® Q columns and a Thermal Conductivity Detector (TCD). Gas production rates were calculated by dividing the obtained gas volume per unit time. The current (mA) and power (mW) generations were derived from the cell voltage (mV) measurements according to Ohm’s laws. The coulombic efficiency (CE) was calculated as described in Logan *et al*. [42].

### DNA extraction and 16S rRNA gene amplicon sequencing

Samples were collected from the MFC feed (swine manure) and anode chambers once during steady-state operation (day 43). The MFCs were opened and 26 g of granular graphite was collected from four different positions (Fig 2). Biofilm was detached from the surface of graphite after incubation of samples in an ultrasonic bath (Selecta, Spain) for 60 s in Phosphate Buffered Saline. Liquid phase was collected after precipitation of graphite granules and cells pelleted by centrifugation at 4,000 rpm. Nucleic acids were extracted from the recovered cell pellets using the Fast DNA® SPIN Kit for soil (MP Biomedicals, USA) according to manufacturer’s instructions. The DNA concentrations were verified using a Qubit^®^ 2.0 Fluorometer (Life Technologies Ltd., Paisley, UK).

The bacterial 16S rRNA gene amplicon sequences were obtained using the bTEFAP method by 454GL FLX technology at the Research and Testing Laboratory (http://www.researchandtesting.com) [43] using primer set 341F-907R [44] modified to contain a 454 FLX Titanium Lib adapter. Sequence denoising, trimming and Operational Taxonomic Units (OTU) assignments (97%) were performed using QIIME (Quantitative Insights Into Microbial Ecology) pipelines [45]. Details of data processing and analysis, together with the calculation of alpha and beta-diversity indices of the bacterial communities at the different sampling positions, are described in the Supplementary Methods.

Core communities were defined for both MFCs using QIIME. OTUs consistently found in at least 3 of 4 samples were selected as members of the core community [46]. Beta-diversity indices were used to analyse differences between bacterial communities according to sampling points or MFCs. Clustering of samples was performed on the basis of the weighted UNIFRAC pairwise distance matrices and visualized as a dendogram [47]. Weighted UNIFRAC distances were calculated and used for the jackknife-resampling analysis. Dendograms of either sample distributions or OTU phylogenies generated in QIIME were visualized in the Interactive Tree of Life software [48].

### Quantitative analysis of bacterial abundance

Bacterial 16S rRNA gene abundance was quantified by quantitative PCR (qPCR) as previously described [49]. Reactions were performed in a 7500 Real Time PCR system (Applied Biosystems, USA) using the SYBR Green PCR Master mix. Standard curves were obtained using serial dilutions (10^2^ to 10^7^ copies) of linearized plasmids. Inhibition tests were performed for each sample. Sample dilution was applied when necessary to avoid inhibition of PCR. Samples for the analysis of bacterial abundances were collected from the two MFC configurations at the four sampling points during steady state conditions. Sampling and quantification of bacterial abundance was performed after applying swine manure to the MFC at three OLRs (10.5 ± 0.7 kg COD m^-3^ d^-1^, 5.3 ± 1.4 kg COD m^-3^ d^-1^, 0.7 ± 0.1 kg COD m^-3^ d^-1^). For all samples, qPCR analyses were performed twice.

### Statistical analysis

All statistical analyses were performed using SPSS for Windows 19.0 (SPSS®, IBM). The ANOVA test was applied for the chemical and electrochemical values obtained from the MFC effluents. Bacterial abundances were related to fluid dynamics by Pearson’s correlation test. The Welch and Games-Howell post hoc tests were used to identify differences in abundance between sample positions and MFCs. Significant differences among whole bacterial community compositions were analysed for the previous variables using the non-parametric ANOSIM method.

### Sequence data submission

The sequences presented in this study have been submitted to the GenBank database with BioProject accession number PRJNA302844.

## Results

### Assessment of MFC performances

**Fig 3:**
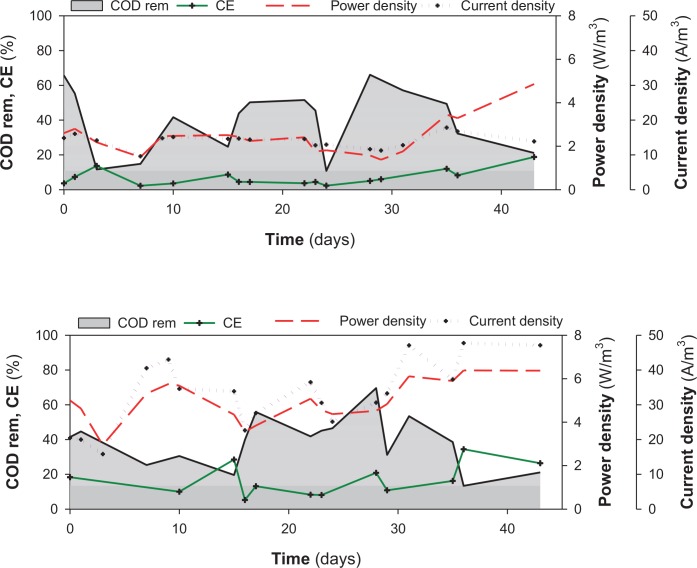
Time course of organic matter removal (COD rem) and electrical performance indicators (CE- coulombic efficiency, Current density and Power density) during the experimental period (43 days) in A) Ref-MFC and B) MPPT-MFC.

The two MFCs behaved similarly in terms of their organic matter removal rate (ORR) and no significant differences were found at an OLR of 10.5 ± 0.7 kg _COD_ m^-3^ d^-1^ (ANOVA test, *p* = 0.61). In both systems, the COD removal efficiency ranged from 36 to 38% (Table 1, Fig 3). The methane (CH_4_) flow rate in Ref-MFC was 10-fold higher than the carbon dioxide (CO_2_) flow rate (48 mL CH_4_ d^-1^ versus 5 mL CO_2_ d^-1^). The ratio between the CH_4_ and CO_2_ flow rates decreased to 2 in the MPPT-MFC primarily due to an increase in CO_2_ production (17 mL d^-1^). This increment of CO_2_ emissions in the MPPT-MFC suggested a higher efficiency of the exoelectrogenic process. In terms of nitrogen, ammonium removal was negligible along the experimental period. Significant differences (*p*< 0.05) between the two MFCs were found for current and power generation and CE values (Table 1). Energy production from the MPPT-MFC (0.025 kWh m^-3^ at 17% CE) was almost double compared with the Ref-MFC (0.013 kWh m^-3^ at 6% CE).

**Table 1:** MFC performances in terms of organic matter and solid removal, current and power generation, and gas production measured for both reactors (Ref-MFC and MPPT-MFC).

Parameter	Units	Ref—MFC	MPPT—MFC	p_value
OLR	kg_COD_m^-3^d^-1^	9.9±2.5	11.2±2.8	0.15
ORR	kg_COD_m^-3^d^-1^	4.0±2.5	4.4±2.7	0.61
η_COD_	%	38±18	36±16	0.12
η_TSS_	%	66±7	55±21	0.76
η_VSS_	%	64±5	54±20	0.72
R _ext_	Ω	30±0	8±3	0.02
I_density_	A m^-3^	14±2	33±10	0.01
P_density_	W m^-3^	2.5±0.8	5.0±1.0	0.01
CE_CODs_	%	6±3	17±7	0.01
CH_4_	mL d^-1^	48±12	39±17	0.06
CO_2_	mL d^-1^	5±3	17±7	0.06
CH_4_/CO_2_	—	6.6±3.8	2.2±1.8	0.05

Values are expressed as the average ± standard deviation. The last column reports the p-value of the ANOVA test. *S*ignificant differences between the microbial fuel cells (MFCs) were set at *p*<0.05.

### Effect of fluid dynamics on bacterial abundance

The calculated fluid velocity was not uniform within the anode chambers despite the recirculation loop application (Fig 2). The position near the influent and effluent sections of the MFC (sampling positions 4 and 1) presented the lowest flow velocities (0.69 and 0.24 m h^-1^, respectively), whereas the recirculation loop positions (positions 2 and 3) presented 10-fold higher values (6.88 and 7.82 m h^-1^, respectively). The highest shear rate was observed at position 3 (16 s^-1^).

The bacterial 16S rRNA gene abundance in the MPPT-MFC (3.1·10^6^ DNA copies g_graphite_^-1^) was higher compared to the Ref-MFC (6.1·10^5^ DNA copies g_graphite_^-1^), but the difference was not significant when all sampling points were considered together. Small differences in the abundance of 16S rRNA genes were detected for the four analysed positions in the two MFCs. Gene abundances were reduced by 20 to 50% at position 3 compared to the other sampled positions (Table 2). This situation remained constant in additional tests at a lower OLR (5.3 ± 1.4 kg COD m^-3^ d^-1^ for 5 weeks and 0.7 ± 0.1 kg COD m^-3^ d^-1^ for 2 weeks). These results demonstrated that the bacterial abundance distribution in the MFCs was influenced by the fluid velocity and shearing effect.

**Table 2:** 16S rRNA gene abundances (Mean values ± SD, n = 2, technical replicates) at the four sampled positions within the Ref-MFC and MPPT-MFC operated at different organic loading rates (OLR).

Reactor	Position	Ribosomal RNA gene abundance per gram graphite (x10^5^)			OLR effect
		High OLR	Intermediate OLR	Low OLR	
**Ref-MFC**	1	3.31±0.47 ab	2.05±0.80 a	42.60±3.36 a	*p*<0.05
	2	1.30±0.09 a	1.34±0.34 a	23.10±4.41 ab	NS
	3	0.16±0.07 b	0.07±0.06 a	0.12±0.08 ab	NS
	4	1.34±0.44 ab	0.07±0.05 a	5.10±1.02 b	NS
**MPPT-MFC**	1	13.64±1.00 a	0.87±0.13 a	79.70±5.14 a	*p*<0.05
	2	16.20±1.16 a	1.10±0.02 a	33.4±6.61 bc	*p*<0.05
	3	0.17±0.16 b	11.8±0.23 b	69.4±3.54 ab	*p*<0.01
	4	0.98±0.20 b	1.03±0.03 a	0.89±0.16 c	NS

Lower case letters next to quantification values show homogenous variance groups among the sampled positions according to pair-wise Games-Howell tests for every reactor and OLR condition. Differences of 16S rRNA gene abundances in every position according to changes of OLR for a single sampling point were analysed with a Welch test. High OLR- 10.5 ± 0.7 kg COD m^-3^ d^-1^, Intermediate OLR- 5.3 ± 1.4 kg COD m^-3^ d^-1^, Low OLR- 0.7 ± 0.1 kg COD m^-3^ d^-1^.

### Bacterial community structure

A total of 22,680 sequences were obtained and used for diversity analyses. The number of sequences varied from a minimum of 554 sequences obtained in MPPT-MFC position 1, to a maximum of 4,868 sequences obtained in Ref-MFC position 4. On average, 2,268 sequences were obtained per sample. Valid sequences were clustered into 474 OTUs at a 97% similarity level, but only 221 OTUs contained at least four sequences and were considered for diversity calculations. Each sample was rarefied to 500 sequences for comparisons of alpha and beta diversity. Despite this reduction in sequence number, the species richness of all samples was considered to be enough covered in view of rarefaction curves (S1 Fig). Significant differences in species richness indicators (observed OTUs and Chao1 estimator) were found between swine manure and the two MFC configurations (Table 3). On the contrary, although Shannon’s and phylogenetic diversity (PD) indices were higher in the swine manure (H’ = 4.23±0.39, PD = 7.22±1.13, n = 2) compared to the MFCs samples (Ref-MFC H’ = 2.81±0.47, PD = 3.69±0.98; MPPT-MFC H’ = 3.85±0.39, PD = 4.59±0.39) no significant differences were found. No significant differences in alpha-diversity indicators of the microbial community structures were observed between the two MFC configurations.

**Table 3:** Observed OTUs and alpha diversity indices, Chao1, Shannon (H’) and phylogenetic diversity (PD) diversity indices in swine manure (SM), Ref-MFC (Ref_1 to Ref_4) and MPPT-MFC (MPPT_1 to MPPT_4).

Samples	Observed OTUs	Chao1	Shannon (H')	PD
SM1	80.7	102.9	5.02	8.37
SM2	61.2	93.2	3.44	6.09
**Mean ± SD**	**70.95±9.75**	**98.03±4.85**	**4.23±0.39**	**7.22±1.13**
Ref_1	27.5	48.1	2.38	2.90
Ref_2	26.7	36.2	2.53	3.08
Ref_3	45.1	68.2	3.60	5.36
Ref_4	30.2	49.7	2.75	3.44
**Mean ± SD**	**32.37±7.46**	**50.57±11.43**	**2.81±0.47**	**3.69±0.98**
MPPT_1	40.9	41.1	4.50	4.75
MPPT_2	39.0	45.4	3.74	4.99
MPPT_3	34.6	40.6	3.74	3.94
MPPT_4	38.1	54.0	3.44	4.69
**Mean ± SD**	**38.15±2.28**	**45.27±5.39**	**3.85±0.39**	**4.59±0.39**
*Pair-wise comparisons*				
**SM-Ref**	*p* = 0.035	*p* = 0.002	NS (*p*>0.1)	NS (*p* = 0.097)
**SM-MPPT**	*p* = 0.019	*p* = 0.030	NS (*p*>0.1)	NS (*p* = 0.082)
**Ref-MPPT**	NS (*p*>0.1)	NS (*p*>0.1)	NS (*p* = 0.079)	NS (*p*>0.1)

Indicated values for each sample are mean values of ten rarefied samples containing 500 sequences. Pair-wise differences of alpha diversity indices according to Feed (SM) or MFC Type were calculated using a t-test. Significance level was set to *p*<0.05. NS- not significant.

**Fig 4:**
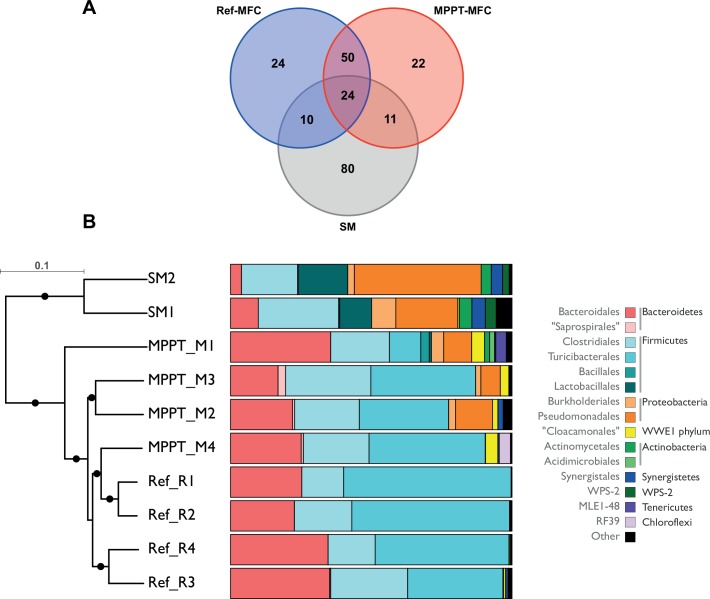
**A)** Venn diagram of the OTU distribution in swine manure and the two MFC configurations (MPPT and Ref). **B)** Clustering of samples based on weighted Unifrac measures (rarefied at 500 seqs per sample) calculated after phylogenetic reconstruction of detected OTUs. Black dots in the dendrogram show nodes at bootstrap supported levels above 80%. Bar charts show the relative abundance of main bacterial groups (Orders) found in each of the samples. Phyla representing less than 1% of the sequences in a sample have been grouped as Others.

Representative sequences for the 221 OTUs were aligned and taxonomy assigned down to the species level when possible. Taxonomy assignments were performed using the Greengenes reference database (v13.8). The swine manure microbial community was primarily composed of bacteria from the phylum *Proteobacteria* (41.8%, OTU 3, showing a high similarity to *Pseudomonas* spp. being the dominant genus) and *Firmicutes* (38.9% of sequences). The bacterial community in Ref-MFC was enriched with bacteria from the phylum *Firmicutes* (69.2% of sequences) and *Bacteroidetes* (29.8%) at all sampling positions, whereas *Proteobacteria* were present at very low relative abundance (less than 1%). In contrast, members of the latter group were consistently found in the MPPT-MFC samples and accounted for almost 7% of the sequences. However, those sequences annotated as *Pseudomonadaceae* found in MPPT-MFC (OTU 12) differed from those found in swine amnure (OTU 3). Members of the candidate division WWE1 (Waste Water of Evry 1) were consistently found at all positions in the MPPT-MFC and accounted for almost 4% of sequences, but rarely occurred in the Ref-MFC and swine manure. OTU 0 (*Turicibacteraceae*), OTU 1 (uncultured p-2534-18B5 gut group), and OTU 2 (*Porphyromonadaceae*) were found at the highest frequencies (35.7% of sequences) and appeared almost exclusively in the MFC samples. Conversely, OTU 3 (*Pseudomonadaceae*) and OTU 6 (*Carnobacteriaceae*) showed a higher relative abundance in the swine manure compared to the MFC biofilms. Only, twenty-four out of 221 OTUs were shared for the three sample types (Fig 4A). The number of shared OTUs increased to 74 when the MFC samples were considered independently.

A reconstructed phylogenetic tree was used to calculate differences in community compositions between sample types according to weighted Unifrac metrics of jacknifed subsamples (500 sequences each). Community structures at the OTU and genus levels were tested for homoscedasticity using betadisper (F = 3.08, *p* = 0.115 for OTU data; F = 1.93, *p* = 0.224 for genus level data). Distances to centroid of the four sampling points revealed significant differences in the bacterial community structure among MFC types (S2 Fig). The microbial community structure of the two MFC types was essentially different from the community structure found in the swine manure even at higher taxonomic levels (Fig 4B). Differences among sample groups (SM, Ref-MFC and MPPT-MFC) were tested using ANOSIM without any transformation of data. Results confirmed significantly different bacterial community structures between the feed (SM) and MFC biofilms (R = 0.688, *p* = 0.01). Differences between the two MFC types were lower (R = 0.385, *p*>0.031). In order to test if variability of microbial communities within each MFC type (intra-group comparisons) was lower than variation among the two MFC types (inter-group comparisons), Bray-Curtis similarity indices were calculated pairwise and compared (S3 Fig). As expected, Ref-MFC yielded a homogeneous low variability group for all intra-group pair-wise comparisons. On the contrary, MPPT-MFC comparisons were highly variable and no significant differences in Bray-Curtis similarity indices were found when compared to inter-group comparisons. High variation in similarity indices in the MPPT- MFC type was due to differences observed in the sample close to the influent (M4).

### The MFC core microbiomes

**Fig 5:**
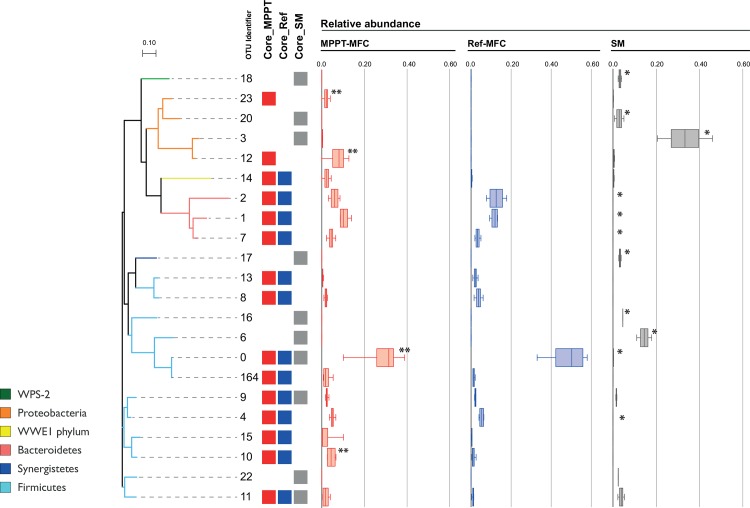
Phylogenetic relationship of OTUs (97% similarity level) belonging to core communities of SM, Ref-MFC, and MPPT-MFC biofilm samples. Members of core communities for each sample type are shown as coloured squares next to OTU identifier. Box plots show the relative abundance of OTUs in swine manure (grey), Ref-MFC (blue), and MPPT-MFC (red). * indicate OTUs showing significant differences on relative abundances between SM and MFCs. **indicate OTUs showing significant differences on relative abundances between Ref-MFC and MPPT-MFC.

OTUs detected at relative abundances higher than 0.5% were considered to estimate the core community of SM, Ref-MFC and MPPT-MFC. Members of the core community of SM were considered if found in the two samples analysed. In the case of the two MFCs, core members were identified as those OTUs found in a minimum of three sampling points within each MFC. The core community common for the SM and both MFCs was limited to three OTUs (OTU 0, 9 and 11), all belonging to the *Firmicutes*. Seven additional OTUs were found exclusively in swine manure. Ten extra OTUs were simultaneously defined as members of the core community of the two MFC configurations. Only two OTUs, the *Proteobacteria* OTU 12 (7.2% of sequences in MPPT-MFC) and 23 (2.0%), were found exclusively in MPPT, revealing some specificity between MFC configurations (Fig 5, S2 Table). No *Proteobacteria* were found and selected as members of the core community in the Ref-MFC.

Relative abundances of OTU 0 (*Turicibacter* sp.), OTU 4 (*Alkaliphilus* sp.) and OTUs 1, 2 and 7 (*Bacteroidetes*) were significantly higher in the two MFCs compared to the SM, suggesting an implication of these bacteria on electrogenesis. The four OTUs accounted for approximately the 80% and 50% of Ref and MPPT-MFCs sequences, respectively. If the two MFC configurations were compared, OTU 10 (*Sedimentibacter* sp.), OTU 12 (*Pseudomonas* sp.) and OTU 23 (uncultured *Oxalobacteraceae*) were enriched in the MPPT-MFC (n = 4, t-test, *p*<0.045, False detection rate, FDR< 0.225, S2 Table), whereas OTU 0 (*Turicibacter* sp.) appeared at higher densities in the Ref-MFC compared to MPPT-MFC (*p* = 0.049, FDR = 0.237). Unfortunately, significant differences between the two MFCs could not be confirmed by FDR corrections of *p*-values, most likely caused by the low number of samples per MFC type (n = 4) and sequences per sample (500 seq.).

## Discussion

Both anode chambers achieved similar ORRs (4.2 kg_COD_ m^-3^d^-1^), solid removal efficiencies (approximately 60% VSS) and gas production rates (in terms of CH_4_ and CO_2_). High OLRs such as those used here may have led to competition between the exoelectrogenic microorganisms and other bacteria for organic substrates, causing an accumulation of gaseous compounds. This phenomenon was previously observed in MFCs by Oliveira *et al*. [50] and specifically for methanogenesis, which was shown to decrease the final CE of the system [51]. Min *et al*. (2005) obtained a 27% COD removal efficiency (1.3 Kg_COD_ m^-3^d^-1^) with a CE similar to that obtained by the Ref-MFC (8%) [5]. A later study improved the COD removal efficiency to 60–70% (3.5 Kg_COD_ m^-3^d^-1^) but the CE remained at values lower than 1.5%, indicating the prominent occurrence of side reactions [52]. The current study showed that the application of a variable resistance control in the MPPT-MFC improved organic matter treatment despite the use of a complex organic matrix for degradation, thereby achieving higher organic removal rates and electric performance, incrementing the CE by 40% and doubling the energy production compared to the MFC with fixed external resistance. These differences may be explained by an enhancement of electrons released when optimal resistances for electron transfer were consistently applied. Intermittent electric connection allowed higher current production, since both capacitive and faradaic currents are harvested. The same positive effect has been observed in a series of MFC configurations, using different solid matrices as electrodes, including granular graphite and marine sediment [53,54]. It has been stated that ammonia levels between 2–8 g N-NH_3_ L^-1^, would cause an inhibition of biofilm anaerobic digestion activity, resulting in less biogas production [55,56]. Similar effects could be hypothesized for electrogenic activity. However, the low influent concentrations used in this study (245±40 mg N-NH4+ L^-1^) are unlikely to have an inhibitory effect on the bacterial community.

The key factor for the proper development of MFC technology relies on the formation of a stable electroactive biofilm. In this sense, the MPPT control has been proven to be more effective for the proliferation of exoelectrogenic bacteria [31] and reduces the start-up time for running a MFC at full capacity [33]. Similar results were obtained here, with the MPPT control exhibiting an increase in both the bacterial abundance at the anode (5-fold compared with Ref-MFC) and the current density (33 and 14 mA/m^3^, respectively).

MFC fluid dynamics were shown to affect the mass transfer kinetics, biofilm structure and production of extracellular polymeric substances (EPS) [57]. In the studied anode chambers, fluid dynamics and shear rates influenced the internal biomass distribution, causing high biofilm detachment near the recirculation loop (position 3) compared to the other analysed positions. The fragility of the different biofilm layers on the electrode could explain the easy detachment that occurred at this position; this finding was in agreement with Shen *et al*., [58] and Celmer *et al*., [57], who reported that a 65% decrease in the biofilm thickness increased the flow rate from 1.3 to 24 mL min^-1^.

The microbial community in swine manure is versatile and changeable depending on variables such as the pigs’ diets or the sporadic use of antibiotics, which have an impact on the gut microbiota [59]. Uncontrolled differences in the microbial composition of the feed could not be avoided and were recorded in the two swine manure samples. Bacteria found in the influent had a limited influence on the community established within the MFCs. Only six OTUs from the MFC core community were identified in swine manure, and all of them belonged to *Clostridiales* (*Firmicutes*). According to 16S rRNA gene based identifications, the *Firmicutes* found were described as having a fermentative behaviour by Siegert *et al*., [60] and Regueiro *et al*., [61]. Previous studies identified *Firmicutes* (*Clostridia*), *Bacteroidetes* (*Bacteroidia*) and *Proteobacteria* (*Gammaproteobacteria*) as the main phyla in swine manure [62]. A recent study suggested a possible exoelectrogenic role for *Clostridium* bacteria because they were detected at a high amount within the exoelectrogenic biofilm of a MFC treating swine manure [26].

According to the OTU-based analysis, the MFC microbiome varied significantly compared to the influent swine manure. Although *Firmicutes* and *Bacteroidetes* also appeared as the dominant phyla in the MFC biofilms, the enrichment of specific OTUs suggested a number of bacteria putatively implicated in exoelectrogenesis. Among these, OTU 0 (*Turicibacter* sp.), OTU 1 (uncultured *Bacteroidetes*), and OTU 2 (*Parabacteroides* sp.) occurred at relative abundances higher than 10% in both of the MFC configurations. On the contrary, *Proteobacteria* were drastically reduced in the anode chambers and were found only in the MPPT-MFC samples. Microbial communities of MFC anodes are usually composed of different bacterial species from which electricity generation capabilities has not been described, but may be hypothesized from comparisons of microbiome structures in selective experimental conditions. The dominance of *Firmicutes* was detected in anode reactors treating swine manure in a previous study of our group [26]. The most abundant phylotype within the two MFCs was identified in base of the partial 16S rRNA sequence as *Turicibacter* sp. (OTU 0), which was barely represented in swine manure samples. *Turicibacter* spp. have been previously found in pig waste, using cultivation-independent molecular analyses [62]. Under strict anaerobic conditions, lactate is the main fermentation product from carbohydrates for *Turicibacter* spp. [63]. Unfortunately, no exoelectrogenic activity has been described for this species. Uncultured p-2534-18B5 gut group (OTU 1) and *Parabacteroides* sp. (OTU 2) were also found at high relative abundances in MFCs. OTU 1 can be related to intrinsic gut microbiota [64], whereas species with a 16S rRNA sequence similar to that of OTU 2 was involved in current generation [65].

Most known exoelectrogenic bacteria fall within the *Proteobacteria*, which have been detected as dominant members of the bacterial community in MFCs treating simple substrates, such as acetate and glucose [66][67][68], and wastes from industrial sources [69][19], revealing a substrate effect on dominant putative exoelectrogenic bacteria. *Proteobacteria* were in competitive disadvantage relative to *Firmicutes* under the experimental conditions applied in this study, as this was most likely related to the presence of highly recalcitrant components of the influent organic matter [70].

The effect of the MPPT control on the bacterial community structure was analysed by comparing the microbiome core communities and related to the increment of current density. The core community of MPPT-MFC contained three different OTUs that appeared at significantly higher relative abundances compared to Ref-MFC. Interestingly, the only OTU found at higher abundances in Ref-MFC was OTU 0, thus questioning its implication in exoelectrogenesis since significantly lower CE was found for Ref-MFC. More likely, *Turicibacter*-related species may be implicated in heterotrophic degradation of organic matter, probably through a fermentation process. However, additional molecular analyses, including shotgun metagenome and metatranscriptome sequencing, will be necessary to identify putative exoelectrogenic bacteria in MFCs based on functional capacity and activity and confirm the previous hypothesis.

*Sedimentibacter* spp. (OTU 10), *Pseudomonas* sp. (OTU 12) and an uncultured *Oxalobacteraceae* (OTU 23) were significantly enriched in the MPPT-MFC. Based on the analysis of 16S rRNA gene similarities, *Sedimentibacter* related species were identified in the core community of MFC systems with high power generation capabilities together with *Geobacter*, *Aminiphilus*, *Acetoanaerobium*, and *Spirochaeta* [71]. Although this was not proven experimentally with activity analyses, the enrichment of these bacteria at higher abundances is likely related to their exoelectrogenic role. Exoelectrogenic capacity has also been proven for some gammaproteobacteria, including *Pseudomonas* species [72].

The MFC microbial community diversity and abundance were studied in relation to the external resistance control and fluid dynamics. The bacterial community from the studied MFCs was more efficient at treating the complex organic matter than the community reported in previous studies. The application of a MPPT electric control in MPPT-MFC resulted in a 5-fold higher bacterial abundance compared to the Ref-MFC and doubled energy production and CE. The adopted electric condition (MPPT *vs* fixed resistance) was more relevant than the fluid dynamics in shaping the MFC microbiome. The MFC core community was primarily composed of the fermentative *Turicibacter* genus. The MPPT control was able to select specific OTUs potentially harbouring higher exoelectrogenic capacities compared to the fixed resistance system. *Sedimentibacter* and gammaproteobacteria were among the most abundant phylotypes that being a member of the core community were enriched in the MPPT-MFC compared to Ref-MFC. Hence, it is likely that these organisms may be related to the extra electricity production in the MPPT-MFC. The optimization of the MFC systems together with the comprehension of the bacterial communities responsible for the internal processes will enable the implementation of MFC technology for *in situ* swine manure treatment. For this reason, future studies could focus on the physical relationship of the dominant taxa with the electrode (fluorescent in situ hybridization analyses) and the identification of active members of the MFC-associated community (shotgun metatranscriptome sequencing).

## Supporting Information

S1 Fig Rarefaction curves of observed OTUs (species) at 97% similarity level for swine manure (SM1 and SM2), Ref-MFC (R1, R2, R3 and R4) and MPPT-MFC (M1, M2, M3 and M4). Sequence subsampling at different number of sequences was performed ten times. Mean values are shown.
(DOC)

S2 Fig Box plot graph showing the distances to centroid of microbial communities determined at a genus level (L6). n = 4 for Ref-MFC and MPPT-MFC, and n = 2 for Swine Manure (SM).
(DOC)

S3 Fig Box plot showing dispersion of Bray-Curtis similarity indices of OTU distribution in Ref-MFC (n = 4) and MPPT-MFC (n = 4) reactor types. All pair-wise combinations of data-points have been organized as Inter-group or Intra-group comparisons and shown as individual points using different colours.
(DOC)

S1 File Supplementary Methods. (DOC)

S1 Table Main characteristics of swine manure. The values are presented as average ± standard deviation (n = 7). n.d. not detected. * Acetic acid and Propionic Acid were the only VFA identified and detected above LOD.
(DOC)

S2 Table Relative abundances of members of the core community in the two MFC types (Ref and MPPT). Core community members were limited to those OTU that were found in at least three of the four samples in the MFC. Differences were assayed using a t-test. FDR (False Detection Rate) correction of p_value. n.d. not detected. NS- not significant (p values >0.1).
(DOC)
