# Supplementary figures and images for: External Resistances Applied to MFC Affect Core Microbiome and Swine Manure Treatment Efficiencies
Source: PLoS One. 2016 Oct 4;11(10):e0164044. doi: 10.1371/journal.pone.0164044 (PMC5049776; doi:10.1371/journal.pone.0164044)

**
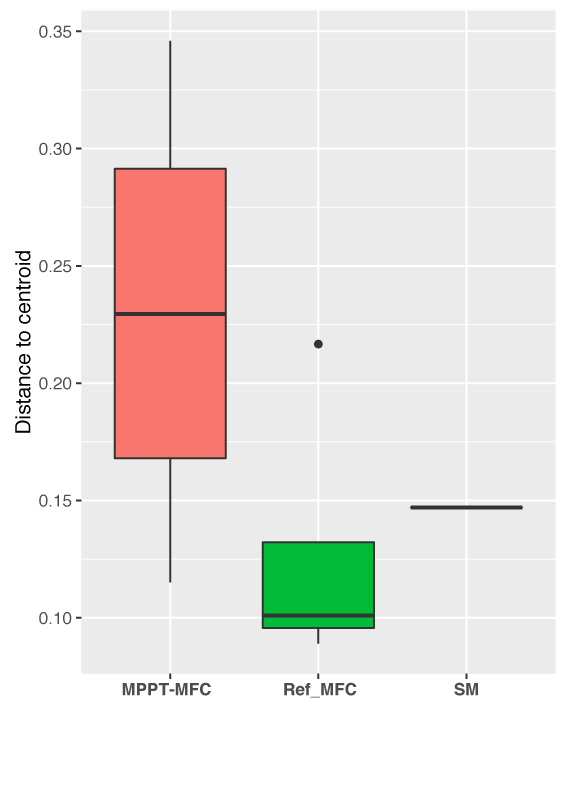
**

Supplement: S2 Fig — n = 4 for Ref-MFC and MPPT-MFC, and n = 2 for Swine Manure (SM). (DOC) [file pone.0164044.s002.doc]

**
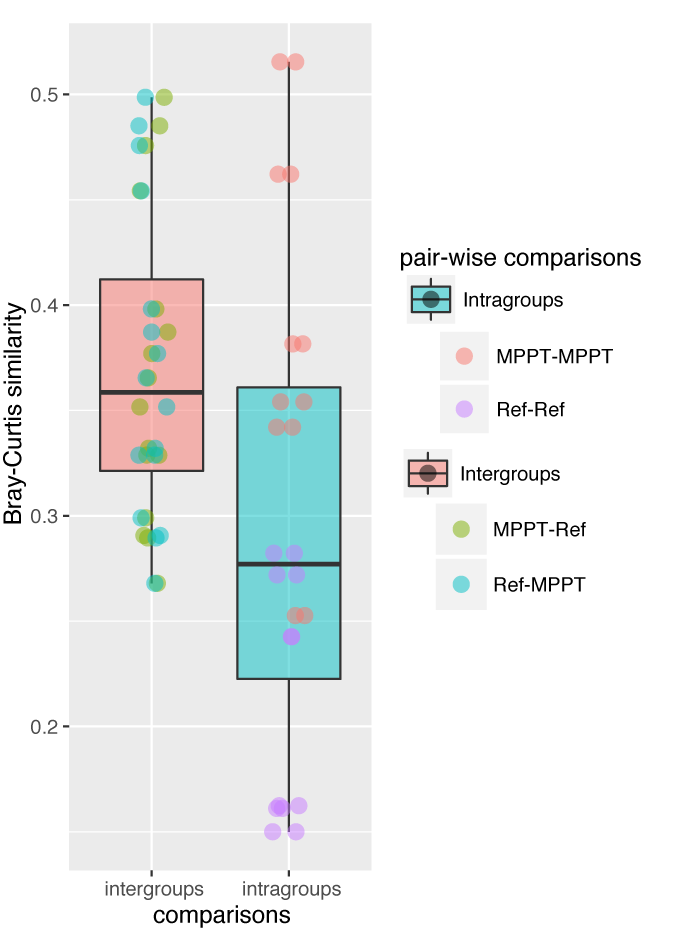
**

Supplement: S3 Fig — All pair-wise combinations of data-points have been organized as Inter-group or Intra-group comparisons and shown as individual points using different colours. (DOC) [file pone.0164044.s003.doc]
