# Supplementary material for: External Resistances Applied to MFC Affect Core Microbiome and Swine Manure Treatment Efficiencies
Source: PLoS One. 2016 Oct 4;11(10):e0164044. doi: 10.1371/journal.pone.0164044 (PMC5049776; doi:10.1371/journal.pone.0164044)
Supplement: S1 File — (DOC) [file pone.0164044.s004.doc]

**Supplemental Materials and methods**

**External resistances applied to MFC affect core microbiome and swine manure treatment efficiencies**

Anna Vilajeliu-Pons^1^, Lluis Bañeras^2*^, Sebastià Puig^1^, Daniele Molognoni^3^, Albert Vilà-Rovira^1^, Elena Hernández-Del Amo^2^, Maria D. Balaguer^1^ and Jesús Colprim^1^

^1^ LEQUiA, Institute of the Environment, University of Girona, Girona, Spain.

^2^ Molecular Microbial Ecology Group, Institute of Aquatic Ecology, University of Girona, Girona, Spain

^3^ Department of Civil Engineering and Architecture (D.I.C.Ar.), University of Pavia, Pavia, Italy

* Corresponding author:

E-mail address: lluis.banyeras@udg.edu (L. B.)

- 1. *Composition of the cathode feed*

The cathode was fed with an oxygen-saturated mineral medium with the following composition: 122 mg L^-1^ NaHCO_3_, 7.6 mg L^-1^ NH_4_Cl, 300 mg L^-1^ NaH_2_PO_4_·2H_2_O, 1.4 mg L^-1^ CaCl_2_, 9 mgL^-1^ MgSO_4_·7H_2_O, 1.3 mg L^-1^ KCl, 150 mg L^-1^ KH_2_PO_4_·2H_2_O and 0.01 mL L^-1^ microelements solution.

*1.2* *MFCs configuration and operation*

Details of MFCs design and operation are described in the study of Molognon*i et al*. (2014) and consisted (each one) of an anode and a cathode placed on the opposite sides of a single methacrylate rectangular chamber. The anode and cathode chambers were filled with granular graphite (model 00514, diameter 1.5-5 mm, EnViro-cell, Germany), which decreased the volumes to 370 ± 10 mL net anodic compartment (NAC) and 410 ± 10 mL net cathodic compartment (NCC) respectively. The electrodes were previously washed in 1 M HCl and 1 M NaOH to remove possible metal and organic contamination. Two thinner graphite rods electrodes (250 x 4 mm, Sofacel, Spain) were introduced in each chamber to allow an external electrical connection to the system. An Anion Exchange Membrane (AMI-7001, Membranes International Inc., USA) was placed between the anode and cathode frames.

The swine wastewater was continuously fed to the anode at a flow-rate of 1.5 L d^-1^. Internal recirculation loops (170 L d^-1^) in each compartment maintained well-mixed conditions and minimized concentration gradients. The temperature of the system was kept constant at 21 ± 1 ºC. The anode potential of each MFC was monitored with an Ag/AgCl reference electrode (+197 mV vs Standard Hydrogen Electrode, model RE-5B, BASI, United Kingdom).

*1.3 Electrochemical analysis calculations*

The OLR was calculated as the total organic matter concentration (COD) divided by the hydraulic retention time (HRT). The organic removal rate (ORR) was determined as the difference between influent and effluent OLRs. The organic matter removal efficiency (ƞCOD) was calculated as the ratio between ORR and OLR.

The total suspended solid (TSS) and volatile suspended solid (VSS) removal efficiencies (ƞTSS/ ƞVSS) were determined as the ratio between solids removal and solids influent concentrations.

Energy production was calculated as the power (P) divided for the flow rate, where the power production is the product of the voltage by the intensity. Coulombic efficiency (CE) was calculated as in Loga*n et a*l. (2006).

*1.4 Sequence trimming and processing with QIIME*

Raw sequence reads were trimmed to 200 bp and quality-filtered using the split-libraries.py implemented in QIIME (Quantitative Insights Into Microbial Ecology) [3]. High quality sequences were checked for chimeras using UCHIME and distributed into operational taxonomic units (OTUs) at a 97% level using either the UPARSE [4], or the UCLUST algorithms. The latter was used to obtain a closed reference sequence file and predict the metagenome. Representative sequences for each OTU cluster were identified and assigned taxonomy with reference to the Greengenes 16S rRNA gene database (release May 2013). OTU tables containing read counts and taxonomic assignments were generated using the script make_otu_table.py. OTUs containing less than 4 sequences in the whole dataset were removed to avoid potential effects of spurious diversity due to sequencing errors. When necessary, identification of relevant OTUs was done after a BLAST search (NCIB) of the obtained representative sequences. Environmental sequences databases were excluded from BLAST searches except when needed (identification of uncultured microbial groups).

*1.5 Structure of the communities*

Community alpha diversity indices, Shannon’s (H’) and Phylogenetic diversity (PD) indices, and maximum richness estimator (S_Chao_) were calculated after rarefying the number of sequence reads in each sample to the lowest value obtained (sample with the lowest number of reads was 503 sequences; Figure S1). Community subsets were calculated at least ten times and mean values for diversity indices estimated. Differences among them were analysed using either parametric or non-parametric tests implemented in group_significance.py in QIIME. Differences were assayed for sample groups according to position of sampling point within the MFC, shear rate due to flow increases, and MFC type. Significant differences were set at p<0.05 level.

Differences on the complete microbial community structures, both at different sampling points and MFC configurations, were inferred from beta-diversity measures. Clustering of samples were performed on the basis of the weighted or un-weighted UNIFRAC pairwise distance matrices and visualized either as a dendogram or a Principal Coordinates plot. UNIFRAC distances were calculated and used for jackknife-resampling analyses with subsampled communities of 500 members.

Significant differences in OTU abundance between the two MFC configurations were analysed using either parametric or non-parametric t-tests implemented in QIIME, and based on rarefied OTU tables for each sample.

**Bibliography**

1. Molognoni D, Puig S, Balaguer MD, Liberale A, Capodaglio AG, Callegari A, et al. Reducing start-up time and minimizing energy losses of Microbial Fuel Cells using Maximum Power Point Tracking strategy. J Power Sources. 2014;269: 403–411.

2. Logan BE, Hamelers B, Rozendal R, Schröder U, Keller J, Freguia S, et al. Microbial fuel cells: methodology and technology. Environ Sci Technol. 2006;40: 5181–5192.

3. Caporaso JG, Kuczynski J, Stombaugh J, Bittinger K, Bushman FD, Costello EK, et al. QUIIME allows analysis of high-throughput community sequencing data. Nat Methods. 2010;7: 335–336.

4. Edgar RC. UPARSE: highly accurate OTU sequences from microbial amplicon reads. Nat Methods. 2013;10: 996–998.
