# Supplementary material for: External Resistances Applied to MFC Affect Core Microbiome and Swine Manure Treatment Efficiencies
Source: PLoS One. 2016 Oct 4;11(10):e0164044. doi: 10.1371/journal.pone.0164044 (PMC5049776; doi:10.1371/journal.pone.0164044)
Supplement: S1 Table — The values are presented as average ± standard deviation (n = 7). n.d. not detected. * Acetic acid and Propionic Acid were the only VFA identified and detected above LOD. (DOC) [file pone.0164044.s005.doc]

|  | **Swine manure** | **Units** |
| --- | --- | --- |
| **pH** | 7.9±0.6 | - |
| **Conductivity** | 2.7±0.3 | mS cm^-1^ |
| **COD _Total_** | 2330±620 | mg COD L^-1^ |
| **COD _Soluble_** | 1330±316 | mg COD L^-1^ |
| **BOD_5 Total_** | 895±220 | mg BOD L^-1^ |
| **BOD_5 Soluble_** | 560±140 | mg BOD L^-1^ |
| **VFA** | 13±7 | mg C L^-1^ |
| **TKN** | 280±50 | mg N-TKN L^-1^ |
| **NH_4_^+^** | 245±40 | mg N-NH_4_^+^ L^-1^ |
| **NO_2_^-^** | n.d | mg N- NO_2_^-^ L^-1^ |
| **NO_3_^-^** | n.d | mg N- NO_3_^-^ L^-1^ |
| **TSS** | 600±450 | mg TSS L^-1^ |
| **VSS** | 545±380 | mg VSS L^-1^ |
