# Supplementary material for: External Resistances Applied to MFC Affect Core Microbiome and Swine Manure Treatment Efficiencies
Source: PLoS One. 2016 Oct 4;11(10):e0164044. doi: 10.1371/journal.pone.0164044 (PMC5049776; doi:10.1371/journal.pone.0164044)
Supplement: S2 Table — Core community members were limited to those OTU that were found in at least three of the four samples in the MFC. Differences were assayed using a t-test. FDR (False Detection Rate) correction of p_value. n.d. not detected. NS- not significant (p values >0.1). (DOC) [file pone.0164044.s006.doc]

|  |  | **OTU ID** | **% Rel. Abund. *** | | |  | **SM vs MFC** | |  | **Ref vs MPPT** | |
| --- | --- | --- | --- | --- | --- | --- | --- | --- | --- | --- | --- |
|  |  |  | **SM** | **Ref-MFC** | **MPPT-MFC** |  | ***p* value** | **FDR corrected *p*** |  | ***p* value** | **FDR corrected *p*** |
| ***Bacteroidetes*** | *Parabacteroides* | **2** | n.d. | 12.6±4.6 | 5.9±2.4 |  | 0.027 | 0.075 |  | 0.054 | 0.238 |
|  | Uncultured *p-2534-18B5* | **7** | n.d. | 3.3±1.3 | 4.4±1.8 |  | 0.014 | 0.047 |  | >0.1 | >0.3 |
|  | Uncultured *p-2534-18B5* | **1** | n.d. | 11.6±1.9 | 10.7±2.5 |  | 0.000 | 0.002 |  | >0.1 | >0.3 |
| ***Firmicutes*** | *Alkaliphilus* | **4** | n.d. | 5.3±1.2 | 5.0±1.3 |  | 0.000 | 0.002 |  | >0.1 | >0.3 |
|  | *Alakalibacculum bacchi* | **16** | 4.6±0.0 | <0.1 | <0.1 |  | 0.000 | 0.000 |  | >0.1 | >0.3 |
|  | *Cryptoanaerobacter phenolicus* | **18** | 3.2±1.1 | <0.1 | n.d. |  | 0.000 | 0.000 |  | >0.1 | >0.3 |
|  | *Sedimentibacter* | **10** | n.d. | 1.2±0.9 | 4.5±2.2 |  | >0.1 | >0.3 |  | 0.042 | 0.224 |
|  | *Syntrophomonas* | **8** | n.d. | 3.7±1.9 | 1.9±0.7 |  | 0.062 | 0.152 |  | >0.1 | >0.3 |
|  | *Syntrophomonas* | **13** | n.d. | 2.1±1.1 | 0.5±0.2 |  | >0.1 | >0.3 |  | 0.061 | 0.227 |
|  | *Tissierella* | **15** | n.d. | 0.4±0.2 | 2.7±2.0 |  | >0.1 | >0.3 |  | >0.1 | >0.3 |
|  | *Thrichococcus pasteurii* | **6** | 14.4±4.9 | <0.1 | <0.1 |  | 0.000 | 0.000 |  | >0.1 | >0.3 |
|  | *Turicibacter* | **0** | 0.2±0.0 | 47.9±11.3 | 28.0±12.4 |  | 0.011 | 0.039 |  | 0.049 | 0.237 |
|  | *Turicibacter* | **164** | <0.1 | 1.4±0.6 | 2.4±2.1 |  | >0.1 | >0.3 |  | >0.1 | >0.3 |
|  | Uncultured *Clostridiaceae* | **11** | 3.8±2.2 | 1.0±0.5 | 1.9±1.7 |  | 0.092 | 0.208 |  | >0.1 | >0.3 |
|  | Uncultured *Clostridiales* | **9** | 1.6±0.4 | 2.1±0.4 | 2.4±0.7 |  | >0.1 | >0.3 |  | >0.1 | >0.3 |
|  | Youngiibacter fragilis | **22** | 2.4±0.0 | n.d. | n.d. |  | >0.1 | >0.3 |  | >0.1 | >0.3 |
| ***Proteobacteria*** | Simplicispira metamorpha | **20** | 2.9±3.1 | n.d. | n.d. |  | 0.012 | 0.024 |  | >0.1 | >0.3 |
|  | Pseudomonas caeni | **3** | 33.3±18.0 | <0.1 | 0.2±0.2 |  | 0.000 | 0.001 |  | >0.1 | >0.3 |
|  | *Pseudomonas sp.* | **12** | 0.4±0.5 | <0.1 | 7.2±5.4 |  | >0.1 | >0.3 |  | 0.042 | 0.224 |
|  | Uncultured *Oxalobacteraceae* | **23** | 0.1±0.2 | n.d. | 2.0±1.6 |  | >0.1 | >0.3 |  | 0.045 | 0.224 |
| ***WWE1*** | Uncultured *Cloacamonaceae* | **14** | 0.3±0.4 | 0.3±0.2 | 2.3±1.8 |  | >0.1 | >0.3 |  | 0.091 | 0.241 |
| ***Synergistetes*** | Cloacibacillum porcorum | **17** | 3.2±0.8 | n.d. | <0.1 |  | 0.000 | 0.000 |  | >0.1 | >0.3 |
