# Supplementary material for: External Resistances Applied to MFC Affect Core Microbiome and Swine Manure Treatment Efficiencies
Source: PLoS One. 2016 Oct 4;11(10):e0164044. doi: 10.1371/journal.pone.0164044 (PMC5049776; doi:10.1371/journal.pone.0164044)
Supplement: Supplementary file 5 [file pone.0164044.s001.doc]

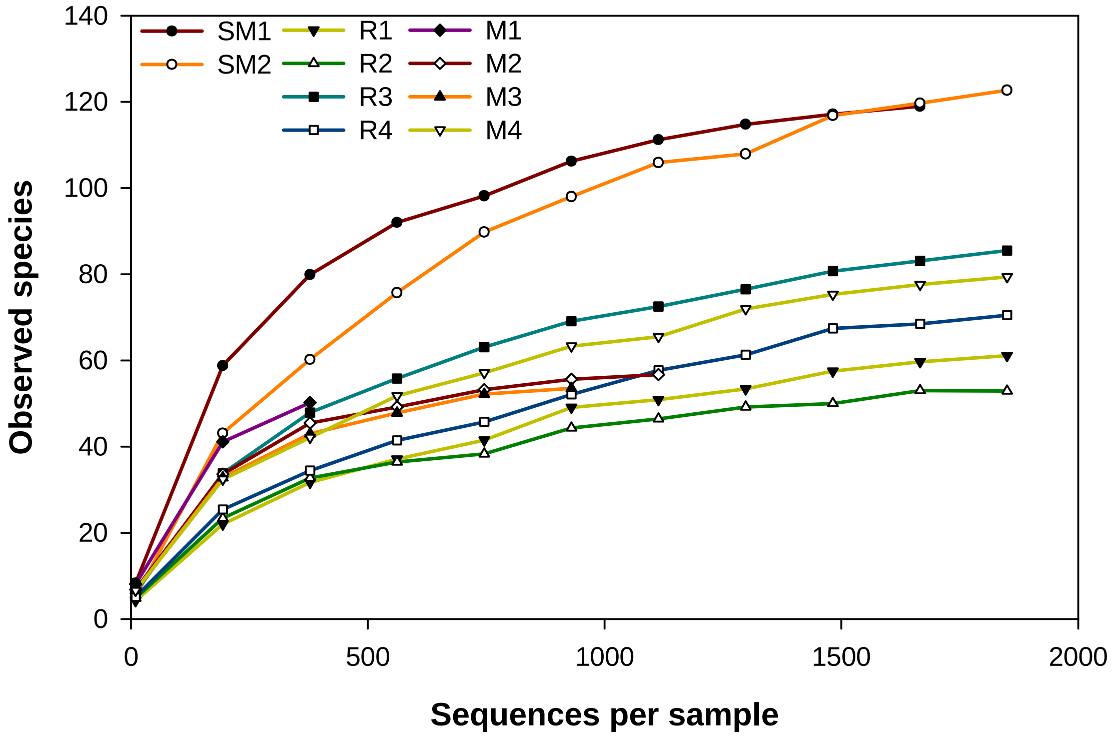

SM1
SM2
R1
R2
R3
R4
M1
M2
M3
M4
Observed species
0
20
40
60
80
100
120
140
0
500
1000
1500
2000
Sequences per sample
